# Biphasic Dose-Response Induced by Phytochemicals: Experimental Evidence

**DOI:** 10.3390/jcm9030718

**Published:** 2020-03-06

**Authors:** Jadwiga Jodynis-Liebert, Małgorzata Kujawska

**Affiliations:** Department of Toxicology, Poznan University of Medical Sciences, 30 Dojazd Str., 60-631 Poznań, Poland; liebert@ump.edu.pl

**Keywords:** cancer, diet, flavonoids, food supplements, hormesis, phytoestrogens, sulforaphane, resveratrol

## Abstract

Many phytochemicals demonstrate nonmonotonic dose/concentration-response termed biphasic dose-response and are considered to be hormetic compounds, i.e., they induce biologically opposite effects at different doses. In numerous articles the hormetic nature of phytochemicals is declared, however, no experimental evidence is provided. Our aim was to present the overview of the reports in which phytochemical-induced biphasic dose-response is experimentally proven. Hence, we included in the current review only articles in which the reversal of response between low and high doses/concentrations of phytochemicals for a single endpoint was documented. The majority of data on biphasic dose-response have been found for phytoestrogens; other reports described these types of effects for resveratrol, sulforaphane, and natural compounds from various chemical classes such as isoquinoline alkaloid berberine, polyacetylenes falcarinol and falcarindiol, prenylated pterocarpan glyceollin1, naphthoquinones plumbagin and naphazarin, and panaxatriol saponins. The prevailing part of the studies presented in the current review was performed on cell cultures. The most common endpoint tested was a proliferation of tumor and non-cancerous cells. Very few experiments demonstrating biphasic dose-response induced by phytochemicals were carried out on animal models. Data on the biphasic dose-response of various endpoints to phytochemicals may have a potential therapeutic or preventive implication.

## 1. Introduction

Compelling data have shown that the consumption of phytochemicals in the form of concentrated supplements can cause adverse health effects if the doses consumed exceed the toxic threshold. However, many reports provide evidence that low doses/concentrations of these compounds have the potential for adverse effects, such as enhancement of the proliferation of tumor cells [1,2]. Various phytochemicals demonstrate nonmonotonic dose/concentration-response termed biphasic dose-response and are considered to be hormetic compounds, for example, resveratrol [2,3] curcumin [4], sulforaphane [1]. The term hormesis described the phenomenon in which a chemical is able to induce biologically opposite effects at different doses; as dose decreases, there are not only quantitative changes in measured responses but also qualitative changes with reference to control and high dose level [5]. Most commonly, there is a stimulatory effect at low doses and an inhibitory effect at high doses [6]. Calabrese et al. [6] characterized two quantitative features of the hormetic response curve: the amplitude of the stimulatory response and the width of the stimulatory dose range. The maximal stimulation of the hormetic response is most typically an increase ranging from 30–60% over control. The stimulatory dose-response is within a 5–100-fold dose range; however, the majority are 5- to 10-fold below the point of response reversal [6,7].

Biphasic, hormetic-like dose-response to various phytochemicals is claimed to be a universal phenomenon. However, a detailed critical survey of source literature does not confirm such an opinion. We revealed that the demarked hormetic nature of some phytochemicals has not been experimentally evidenced. Moreover, the term “hormesis” is often misused and the most common default refers to the identification of hormetic properties exclusively on the basis of low dose effects which is contradictory to the classic definition of hormesis [8].

Phytochemicals are natural components of the diet, food supplements, and medicines, therefore understanding the nonmonotonic response of biological systems to these compounds should receive considerable attention.

Our aim was to present the overview of the reports in which phytochemical-induced biphasic dose-response is experimentally proven. Hence, we thoroughly analyzed every original article found in the process of our literature search and selected those in which the reversal of response between low and high doses/concentrations of phytochemicals for a single endpoint was documented.

We have excluded curcumin from this work since its hormetic properties were recently reviewed elsewhere [4]. As data on the biphasic concentration/dose-response displayed by resveratrol were extensively reviewed in 2010 [2,3], we presented here reports concerning this subject published from 2010 until 2019. We have divided our review into three sections. The first one is dedicated to phytoestrogens because the majority of reports on biphasic concentration-response induced by phytochemicals referred to this group of compounds. Resveratrol deserves a separate section because it “commonly displays hormesis” [2]. The rest of the phytochemicals were discussed in one common section because for such diverse chemicals, no logical criteria for a division into subgroups were found. We limited the area of review to pure compounds; no extracts or juices were considered.

The literature search was conducted in PubMed, Web of Science and Google Scholar databases from 1990 to 2019; the key search terms were “phytochemicals” or “hormesis” or “biphasic dose-response” or “biphasic concentration-response” or “biphasic effect.”

## 2. Phytoestrogens

Phytoestrogens are compounds of plant origin, which chemical structure is similar to 17β-estradiol (E2). Their action is mediated by both α and β subtypes of estrogen receptors (ERs). It has been demonstrated that phytoestrogens may protect against hormone-dependent cancers, for example, breast cancer. Two major soy isoflavones, genistein, and daidzein, are used as an alternative for estrogen replacement therapy because they bind to estrogen receptors and display estrogenic effects [9]. 

**Genistein** (4″,5,7-trihydroxyflavone) (GEN) exerts biphasic effects in various tumor cell lines. A number of studies have shown that genistein induces proliferation of estrogen-dependent MCF-7 cells at low concentrations, below 1 μM, and is cytostatic at higher concentrations, above 10 μM [9,10,11,12,13,14,15,16,17,18,19]. The magnitude of stimulation of cell growth was in a wide range: 10% [15], 20% [12,14,18], 60% [17], 100% [13], and 190% [11]. These findings were confirmed in an animal experiment with MCF-7 cells implanted s.c. in ovariectomized athymic mice. Emerging tumors were about 2-fold larger in the genistein (750 ppm in the diet) treated group as compared to those in the controls [11]. The authors of the above-cited articles concluded that the proliferative effect of GEN in MCF-7 cells is associated with the estrogen receptor pathway, while the effects of higher concentrations were independent of the ER. A similar biphasic effect of GEN on prostate cancer cells PC-3 proliferation was demonstrated. At the concentration 0.5–1 µM genistein caused a 1.5-fold increase in cell number as opposed to >3-fold decrease with 50 µM, compared to vehicle-treated cells. The authors revealed that genistein could stimulate invasion of PC-3 cells via upregulation of osteopontin (metastasis promoter) and subsequent activation of matrix metalloproteinase-9 (MMP-9). The concentration 0.5 –1 µM represents a physiologically achievable level, which might enhance the proliferative and metastatic potential of undiagnosed early-stage prostate cancer via an estrogen- and phosphatidylinositol 3 kinase (PI3K)-dependent mechanism [20]. This suggestion was supported in the experiment with transgenic adenocarcinoma mouse prostate (TRAMP-FVB) mice fed genistein at the dose equivalent to the lower concentration used in the above-mentioned in vitro experiment (250 mg/kg diet) for 8 weeks. The authors observed the progression of prostate cancer by a 16% and 70% increase in the incidence of pelvic lymph node metastases. Administration of the dose 1000 mg/kg diet resulted in a much smaller progression of prostate cancer. However, the high dose did not evoke the opposite effects; hence, this pattern of dose-response cannot be classified as biphasic [20]. The biphasic effect of GEN was also demonstrated in nontumorigenic human prostate epithelial cells, RWPE-1, which express the ERβ receptor [21]. Treatment of the cells with GEN at the concentration of 1.5–12.5 μM increased cell proliferation by 4–58%. The concentrations of 50 µM and 100 µM decreased cell proliferation by 18% and 60%, respectively. Treatment of cells with a model antiestrogen (ICI 182,780) caused inhibition of genistein-induced proliferation. These changes were paralleled by the increase in extracellular signal-regulated kinase (ERK1/2) activity by the lower concentration (about 30%) and a marked decrease (about 95%) after incubation with the higher concentration. The results suggest that GEN modulates RWPE-1 cell proliferation via an estrogen-dependent pathway involving ERK1/2 activation. The effect of GEN on proliferation was examined in benign tumor cells: human uterine leiomyoma (UtLM), and uterine smooth muscle cells (UtSMCs). A low concentration of GEN, ~3.7 μM stimulated the 2-fold proliferation of UtLM cells. Simultaneously the expression of proliferating cell nuclear antigen (PCNA) and the percentage of cells in S phase was increased. This process did not occur in UtSMCs. Higher concentrations (>37 μM) inhibited proliferation, adversely affected morphology, and induced apoptosis in both cell lines. The increased responsiveness observed in UtLM cells could be due to enhanced transactivation of the ER and up-regulation of various transcription factors, growth factor peptides and receptor tyrosine kinases, which have been previously shown to be up-regulated in response to treatment with 17βE2 in UtLM cells [22].

The biphasic effects of GEN on parameters different than the proliferation/viability of cultured cells were also demonstrated [23]. At concentrations 0.1–10 μM GEN stimulated osteogenesis in mesenchymal progenitor cells KS483, as evidenced by the increase in alkaline phosphatase (ALP) activity, nodule formation, and calcium deposition, with the maximal effect at 1 μM (3.3–4.4 fold increase). At concentrations 25 μM and higher, all these parameters were inhibited by 40–90%. Similar stimulatory and inhibitory effects of GEN on bone formation were also shown in mouse bone marrow cell culture. The biphasic effect was also observed for adipogenesis. At low concentrations, 0.1–1 μM, GEN decreased adipocyte number by 85%, while at higher concentrations (>10 μM) it stimulated adipogenesis to a 3.4-fold increase. The authors proposed the mechanism of GEN effects on both parameters. They showed that GEN in addition to its ER affinity at micromolar concentrations binds to and transactivates peroxisome proliferator-activated receptor γ (PPARγ), the transcriptional factor essential for adipogenesis, leading to a down-regulation of osteogenesis and up-regulation of adipogenesis. They pointed out that the balance between ERs and PPAR activation determines the biological effects of genistein. It is well established that ligand activation of PPAR results in inhibition of cell growth and induction of apoptosis. Hence the authors concluded that GEN inhibits the cell growth of cancer cells as evidenced elsewhere [10,11,12,17,18] because of its ability to activate PPAR [23].

The animal experiment supporting in vitro findings related to the proliferative activity of GEN low doses, was reported by Liu et al. [14]. Transgenic erbB-2/neu mice relevant to human breast cancer were given a diet containing a mixture of soy flavones enriched with genistein and daidzein: 211 µg/g diet and 500 µg/g diet. Tamoxifen-associated mammary tumor prevention was significantly reduced (50%) in mice fed the low-dose isoflavone enriched diet. The higher-dose isoflavone diet did not cause such an effect.

**Daidzein** (7,4′-dihydroxyisoflavone) (DAI) affects the proliferation of human breast cancer cells T-47D in a biphasic dose-response pattern. At concentrations ~1–79 μM DAI enhanced cell growth (the maximum effect 150% increase at ~20 μM), whereas the growth was inhibited by 54% at the concentration ~157 μM. The authors suggested that the underlying mechanism might be associated with the levels of cell cycle regulatory protein, p53 [24]. A similar pattern of dose-response was observed in another human breast cancer cell line, MCF-7 which proliferation was stimulated (30% increase) by daidzein at ~1 μM. Concentrations higher than 10 μM caused the inhibition of proliferation, 50% at ~197 μM, and 65% at ~393 μM [25]. In colon cancer cell line LoVo, treated with 0.1–50 µM of DAI, a biphasic effect of the compound tested on proliferation was observed. Concentrations 0.1 and 1 µM stimulated the growth of cells by 10–12%. At higher concentrations (10–100 μM), cell growth was inhibited in a concentration-dependent manner by 5–30%. These concentrations caused cell cycle arrest at the G0/G1 phase, DNA fragmentation, and an increase in caspase-3 activity [26]. Dang et al. [27] investigated the effects of DAI in noncancerous cells, namely mouse bone marrow cells and mouse osteoprogenitor cells KS483, which can concurrently differentiate into osteoblasts and adipocytes. DAI stimulated osteogenesis and decreased adipogenesis at concentrations below 20 μM whereas it inhibited osteogenesis and stimulated adipogenesis at concentrations >30 μM. DAI concurrently activates ERs and PPARs, and the balance between the action of these molecules determines the effect of DAI on both parameters tested [27].

**Quercetin** (QER) (5,7,3,4′-flavon-3-ol) found abundantly in fruit and vegetables displays estrogenic activity and can affect cultured cells’ proliferation in a biphasic manner. Low concentrations of QER, up to 1 μM, caused a marked increase in proliferation of the two human breast cancer cell lines, MCF-7 SH and MCF-7 WT, by 4.2-fold and 2.6-fold, respectively. Concentrations 10 μM and higher led to massive cell death. The authors confirmed that the stimulating effects of QER (not cytotoxic) were ER-dependent [17]. Similar results were reported for the colon carcinoma cell lines HCT-116 and HT-29. High concentrations of QER, above 30 μM and 80 μM, respectively decreased proliferation of both lines. About a 20% increase in proliferation was observed at lower concentrations: 1–30 μM for HCT-116 cells and 1–67 μM for HT-29 cells. Within the concentration range tested only a stimulating effect, up to 100%, for the MCF-7 cells was noted [28]. Incubation of human oral squamous carcinoma cell line SCC-25 with various concentrations of QER also showed a biphasic dose-response. Exposure to 1–10 µM of QER resulted in growth stimulation of cells, whereas the cytotoxic effect was observed at 100 μM of the compound tested [29].

Quercetin was also found to display biphasic concentration-response not linked to its estrogenic activity. A strong stimulatory effect (about 60%) of QER on the cyclooxygenase mediated formation of prostaglandin E2 (PGE2) in murine macrophages RAW 264.7 was observed at physiologically achievable concentrations, 10–100 nM. Higher concentrations (10–100 μM) cause a severe drop in PGE2 content [30]. The authors intended to confirm these findings in the in vivo model. They investigated the effect of QER on plasma PGE2 levels in male Sprague–Dawley rats administered increasing doses of QER, 0.05–5 mg/kg b.w. in single i.v. injection [31]. At lower doses up to 0.3 mg/kg QER stimulated the formation of PGE2 by about 5-fold. Higher doses treatment (40 mg/kg) resulted in the reduction of PGE2 levels; however, the opposite effect, i.e., inhibition of PGE2 formation (as compared to controls) was not observed. Hence, it seems that the described effects in vivo cannot be classified as biphasic ones. A biphasic effect of QER on human basophil activation was reported by Chirumbolo et al. [32]. The authors incubated basophils with the bacterial peptide fMLP and evaluated the up-regulation of two membrane markers: the tetraspan CD63 and the ectoenzyme CD203c, which are commonly used to assess basophil response to external stimuli. QER at concentration ~0.03–0.33 μM increased expression of both markers by 52% and 37%, respectively, whereas ~3–33 μM caused a reduction in expression with the maximum effect observed at the highest concentration tested, 14% and 6% of the control values. The authors suggested that the enhancing effect of low QER concentrations on the activation of basophils might be considered beneficial because of the strengthening inflammatory reaction against invading bacteria [32]. The same authors extended their studies using a similar experimental model [33]. They confirmed the above findings and additionally reported on a biphasic pattern of histamine release from basophils activated by fMLP. Low concentrations of QER 0.03–0.3 µM caused a 2-fold increase in histamine level. The highest concentration tested, 33 µM, inhibited histamine release by 75% as compared to the control. Moreover, the authors suggested the involvement of PI3K in this effect of QER. Contrary to the above-cited results, low concentrations of QER are not beneficial in the context of its potential use in the prevention of allergies [33].

QER has been found to extend lifespan in nematode *Caenorhabditis elegans* in a biphasic dose-response manner. The magnitude of response was rather small but statistically significant. Concentration 100–200 µM caused about a 10% increase in lifespan, whereas treatment with 250 µM decreased lifespan by about 7%. The authors identified several genes putatively involved in QER life-extending action. They concluded that antioxidant/prooxidant properties of QER, modulation of some genes as well as the relocation of energy contributed to the observed biphasic effect on life extension [34].

Quercetin was reported to modulate the activity of model mutagens in biphasic concentration-response mode. The compound stimulated 2-fold the mutagenic activity of AFB1 at concentration 0.06–0.12 mM and inhibited mutagenesis at a lower concentration of 0.006–0.01 mM by about 10%. The authors suggested that the lack of consistency in the observed health effects of various flavonoids might be due to the fact that these compounds or their metabolites can modulate in a different way the activity of enzymes responsible for the activation and detoxication of carcinogens [35]. The biphasic effect of quercetin on the mutagenicity of 2-amino-3, 4-dimethylimidazo [4,5-f]quinoline (MeIQ) using a *Salmonella typhimurium* test was reported by Kang et al. [36]. Mutagenicity was enhanced by quercetin by 50% and 42% at 0.1 µM and 1 µM, respectively, but suppressed by 82% and 96% at 50 µM and 100 µM. The authors claimed that this effect was due to the biphasic concentration-response of CYP1A2 activity to the compound. Its low concentrations stimulated enzyme activity by 10–15%, which resulted in the elevated production of active metabolites of MeIQ. At the highest concentration tested (100 µM) CYP1A2 activity was inhibited by 40%, leading to the decreased mutagenicity of MeIQ [36].

**Biochanin A** (5,7-dihydroxy-4′-methoxyisoflavone) was demonstrated to elicit biphasic dose-response of the proliferation of two cancer cell lines. Human breast carcinoma cells MCF-7 were incubated with biochanin A at concentrations ~0.35–352 μM. At concentrations less than 35 μM cell proliferation was stimulated by 23% as compared to controls; concentrations higher than 106 μM biochanin A inhibited cell growth: by 50% at ~141 μM and by 75% at ~352 μM. A similar biphasic effect was observed for DNA synthesis: concentrations of ~18 μM caused a 180% increase, whereas at ~70 μM DNA synthesis was reduced to 47% of the control value. At concentrations higher than 141 μM, no measurable DNA synthesis was found [37]. Similar findings, although limited to two doses, were reported by Ying et al. [24] who examined the effect of biochanin A on the proliferation of human breast cancer T-47D cell line. Biochanin stimulated cell growth at a concentration ~4 μM by 36% and inhibited growth at ~70 μM by 40%. The level of p53 protein was higher in cells treated with ~70 μM of the compound tested [24].

Natural prenylated flavones characterized by the presence of an isopentenyl group at C-8: **artelastin, artelastocarpin, artelastochromene, and carpelastofuran** demonstrated the biphasic effect on DNA synthesis in MCF-7 cells. At low concentrations of 0.02–2.9 μM, they stimulated DNA synthesis by 130–200% as compared to controls. Concentrations higher than 3.12 μM inhibited cell growth, and DNA synthesis was stopped at a concentration 25 μM. The compounds tested did not stimulate DNA synthesis in estrogen-independent MDA-MB-231 cells, which suggests the involvement of an estrogenic receptor in their proliferative effect [38,39].

Another prenylated flavone, **breviflavone B** also stimulated the proliferation of MCF-7 cells with peak activity at 450 nM (1.9-fold increase). Higher concentrations, 2.2–6.6 μM inhibited the growth of cells and additionally, ERα protein expression, reducing it to about 15% of the control value. This could partially explain a possible mechanism for the observed biphasic effect—proliferative action of breviflavone driven by ERα stimulation was ceased as a result of ERα protein inhibition [40].

The prenylated isoflavonoid **glabridin**, the major isoflavan in licorice root, is an agonist of human ER. Glabridin stimulated the growth of breast tumor cells T-46D over the range of concentrations 0.1–10 μM, reaching the maximum level (about 2-fold of controls) at about 10 μM. Concentrations higher than 15 μM caused abrupt inhibition of cell growth [41].

**Glabrene**, an isoflavene isolated from licorice root, can bind to the human ER with higher affinity than glabridin. The growth of breast tumor cells T-47D and MCF-7 was increased as a result of incubation with increasing concentrations of the compound, 100 nM-10 μM 3.5-fold, and 75% (maximum values), respectively. Concentrations higher than 15 μM inhibited cell proliferation [42].

Mammalian lignan-type phytoestrogens **enterodiol** and **enterolactone** are produced by the action of colon microbiota from plant lignans. Feng et al. [43] reported on the increase (by about 20%) in the viability of human osteoblast-like cells MG-63 incubated with these compounds at a concentration of ~33 μM. Concentrations higher than 333 μM caused a marked decrease in cell viability (about 90%). Similarly, ALP activity (a marker of osteogenic activity) was increased by 35% at concentrations ~33–333 μM and reduced by 40–60% at higher concentrations (3–33 mM). Parallel mRNA levels of osteonectin and collagen I also followed biphasic response [43].

**Isoliquiritigenin** (ISL) (2′,4,4′-trihydroxychalcone) isolated from licorice root is the agonist of ERα. Concentrations of ISL up to 1 μM induce MCF-7 cell proliferation by about 3-fold, whereas concentration 10 μM induced a severe drop in cell number as a consequence of cytotoxicity. The authors confirmed that the ERα–mediated mechanism is involved in ISL stimulated cell proliferation [44]. Kang et al. [45] demonstrated a biphasic effect of ISL on tissue inhibitors of matrix metalloproteinases (TIMPs), which counteract matrix metalloproteinases (MMPs)-mediated tumor invasion. The protein expression of TIMP-2 was elevated in human umbilical vein endothelial cells (HUVEC) exposed to phorbol myristate acetate. Treatment of cells with ISL at concentrations <10 μM caused a further 4-fold increase in TIMP-2 expression, whereas 25 μM ISL suppressed TIMP-2 expression to a level lower by 30% than that in controls. The authors suggested that low concentrations of ISL may increase the therapeutic efficacy of antitumor drugs [45].

**Kaempferol**, one of the common dietary phytoestrogens, induced the proliferation of MCF-7 breast cancer cells at concentrations lower than 1 µM (the maximum effect 4-fold increase). Concentrations higher than 1 µM caused the inhibition of cell proliferation [46].

Generally, the authors of the above-presented reports concluded that the effects of low concentrations of phytoestrogens were mediated by ERs. High concentrations of phytoestrogens may function as estrogen antagonists and inhibit cell growth by competing with estradiol on binding to the ER site [9]. However, many studies revealed that their action at higher concentrations is ER-independent and other molecular targets are involved [9].

The biphasic effect of phytoestrogens on cell proliferation is essential in view of its use as an ingredient of food supplements. There is accumulating evidence that health benefits occur when phytoestrogens are consumed in appropriate quantities. It has been reported that the plasma concentration of GEN is relatively low and less than 40 nM (the level of stimulating cell proliferation) in humans consuming diets without soy. However, it can be much higher, about 40 μM in those who consume large amounts of soy products [47]. The blood serum level of QER from the ingestion of a standard diet varies around 1 μM; the concentration found to enhance cell proliferation. Higher QER concentrations are expected following the ingestion of the QER supplement [17]. The concentration of biochanin A <35 μM which stimulated the cell proliferation in vitro, is within the reported in vivo range (~1–11 μM) in the plasma of humans consuming soy-rich diet [37].

Some authors argue that long term exposure to low levels of phytoestrogens could stimulate the progression of estrogen-dependent tumors [20,28,44]. Hence, dietary recommendations should be considered carefully in women affected by hormone-sensitive breast cancer. In the recently published article, Rietjens et al. [9] presented a comprehensive overview of the health effects of phytoestrogens. Numerous health benefits of these compounds have been reported; however, there is also evidence for their potential adverse effects, e.g., endocrine disruption. The authors claimed that a more refined quantitative risk-benefit should be made to conclude definitely on the health effects.

**Figure 1:**
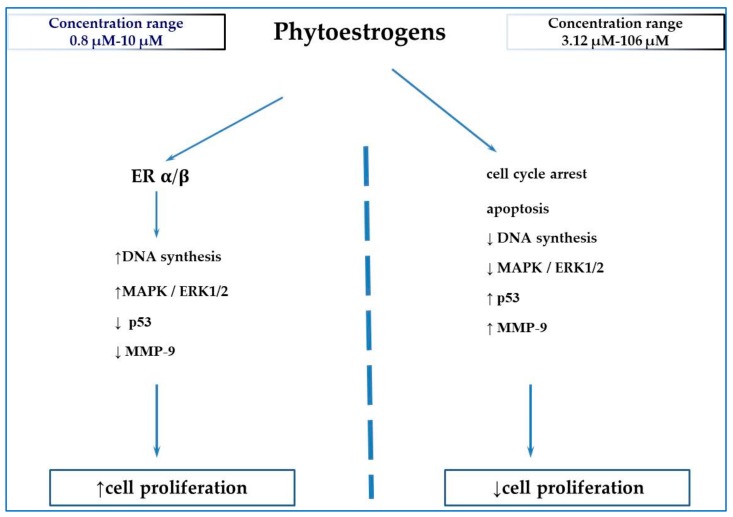
Suggested mechanisms of biphasic concentration-dependent effects of phytoestrogens (on the basis of references cited in the review). ↑ = increase, ↓ = decrease; ER—estrogen receptor; ERK—extracellular signal-regulated kinase protein-serine/threonine kinase; MAPK—mitogen-activated protein kinase; MMP-9—matrix metallopeptidase 9; p53—tumor protein p53.

Data on the biphasic effects of the above-discussed phytoestrogens and suggested mechanisms are presented in Table 1 and Figure 1.

## 3. Resveratrol

As mentioned above, in the current review, we presented data concerning biphasic concentration-response induced by resveratrol and published after 2010.

Biphasic concentration-response to resveratrol has been commonly demonstrated for standard parameters measured routinely in cell culture: viability and proliferation. Plauth et al. [48] found that treatment with a lower concentration of RES moderately increased the viability of several cell lines: neonatal normal human epidermal keratinocytes (NHEK) by 20% (<50 μM); neonatal normal human dermal fibroblasts (NHDF) by 15% (1–300 μM); and HepG2 cells by 15% (1–100 μM). The high concentration of RES (500 μM) markedly reduced cell viability: 75% for NHEC and NHDF, and 40% for HepG2. The authors proposed that the increased fitness of cells treated with low RES concentration is due to the enhanced expression of cellular defense genes, the process triggered by gentle oxidative stress evoked by RES [48].

At 1 μM, 10 μM and 20 μM, RES stimulated the proliferation of neural progenitor cells by 10%, 35%, and 25%, respectively. Higher concentrations, 50 μM and 100 μM decreased cell proliferation by 50% and 65%. A similar relationship was reported for proliferation markers nestin and SOX2. The levels of both molecules were increased by 10–50% in cells incubated with 1 μM, 10 μM and 20 μM. Higher concentrations tested decreased their levels by 20–50%. The authors suggested that enhanced proliferation was mediated by increased phosphorylation of extracellular signal-regulated kinases (ERKs) and p 38 kinases. Higher RES concentrations significantly reduced the activation of these molecules [49].

A similar effect of RES on cell proliferation was observed for colorectal adenocarcinoma cells HT-29. At concentrations, 1–10 μM RES increased about 2-fold a number of cells whereas at 50 μM and 100 μM, the percentage of necrotic and apoptotic cells was reduced by 76% and 90%, respectively. RES-induced cytotoxicity was associated with NADP oxidase activation and increased level of histone ɣH2AX, a marker of DNA damage [50].

Bovine spermatozoa viability was also affected by RES in a biphasic mode. At lower RES concentrations, 1–50 μM, an increase in this parameter by 10–75% was noted. Incubation of cells with 100 and 1000 μM resulted in inhibition of the cell viability by 50% and 65%, respectively. Superoxide anion production in spermatozoa incubated with growing concentrations of RES also displayed biphasic concentration-response mode. Low RES concentrations, 1–50 μM reduced superoxide level by 15–50%; higher concentrations, 100 and 200 μM caused a 40% and 60% increase, respectively, as compared to controls. The consistency between the effects of RES on spermatozoa viability and superoxide production once more confirmed the role of prooxidant RES action in cytotoxicity [51].

RES induced a biphasic effect on DNA synthesis in androgen-sensitive LNCaP cells. At 5 μM and 10 μM RES caused a 2–3-fold increase in DNA synthesis—due to the induction of cells’ entry into S-phase, whereas at >15 μM DNA synthesis was inhibited [52]. Similar effects were observed in rat granulosa cells. RES at 10 μM stimulated thymidine incorporation by 54%, whereas concentrations of 30 and 50 μM decreased this process by 49% and 44%, respectively [53]. The authors of both reports suggested that the unique ability of RES to exert opposing action on two essential processes in cell cycle progression: induction of S phase and inhibition of DNA synthesis is responsible for the described effects.

Guo et al. [54] reported that RES biphasically modulated chromosomal instability (CIN) in human normal colon epithelial cells. At low RES concentrations (0.1–1 μM) basal levels of CIN markers micronuclei (MN) and nucleoplasmic bridge (NPB) were reduced by 17–63%; the most marked decrease was noted at 0.1 μM. The higher RES concentration, 100 μM, increased the MN value by 30% and NPB by 10%. Consistently with the above findings, cell viability was slightly increased (10%) and significantly decreased (35%) when incubated with 0.1 and 100 μM of RES. The authors suggested that the biphasic effect of RES on CIN might be attributed to the regulation of mitotic fidelity through the SAC (spindle assembly checkpoint) pathway which is a major cell-cycle regulatory network controlling chromosome segregation during mitosis [54].

Besides cell viability/proliferation and DNA synthesis, other parameters were also modulated by RES in a biphasic mode. Bosutti et al. [55] investigated the effect of RES (10–60 μM) on C12C12 myoblast and myotube plasticity. Low RES concentration (10 μM) stimulated myoblast cell cycle arrest, migration, and sprouting which were inhibited by 40–60 μM. However, only cell motility displayed biphasic concentration-response. At 10 μM cell motility was enhanced by 38% whereas the number of migrated cells was decreased by 17–70% by increasing concentrations of RES. The authors concluded that low concentrations of RES might promote in vitro muscle regeneration [55].

In a HepG2 cell culture, the high concentration of RES (100 μM) decreased the extracellular level of apolipoprotein M (apoM) by about 35% whereas moderate concentrations (1 and 10 μM) increased 2-fold its extracellular level. ApoM is a carrier and modulator of sphingosine 1-phosphate (S1P), a product of sphingosine kinase (SK), which exerts beneficial effects in cardiovascular diseases [56].

Peltz et al. [57] examined the effects of RES on cell self-renewal and differentiation of human mesenchymal stem cells (hMSCs), which could differentiate into multiple cell types. They demonstrated that at 0.1 μM RES inhibited cellular senescence by 10%, at 1 μM had no effect whereas at 5 and 10 μM the senescence rate was increased by 6% and 15%, respectively, as compared to controls. Despite their small magnitude, the changes were statistically significant. This finding was confirmed in the assay based on beta-galactosidase activity, an indicator of cellular senescence. The number of senescent cells was decreased by treatment with 0.1 and 1 μM by 30% and 50%, respectively. Higher concentrations of RES (5 μM and 10 μM) caused an increase in the number of senescent cells by 40% and 225%, respectively. These findings could be partly explained by the fact that some genes implicated in cell survival (e.g., sirtuins, birc) were upregulated by a lower concentration of RES but inhibited by higher concentrations [57].

The antigenotoxic effects of RES were investigated in HepG2 cells exposed to model mutagen 4-nitroquinoline-N-oxide (4NQO). A slight antigenotoxic effect at concentrations 10, 25, and 50 μM was observed with genotoxic inhibition rate (GIR) 12%, 26%, and 34%, respectively. For concentrations of 100 and 250 μM, the extent of DNA damage was greater than for 4NQO by 33% and 66%, respectively. The highest concentration tested significantly induced apoptosis, hence the authors suggested that the pro-apoptotic effect of RES could, in part, explain the above described biphasic concentration-response [58].

RES demonstrated the concentration-dependent biphasic effect on human natural killer (NK) cells, which play an essential role in tumor identification and surveillance. Cytotoxicity of NK cells was slightly increased by 4% and 6% (statistically significant increase) when incubated with low RES concentrations (1.56 and 3.13 μM). RES concentrations of 25 and 50 μM diminished NK cells cytotoxicity by 29% and 39%, respectively. At 3.13 μM RES was demonstrated to enhance the expression of both TNFγ (by 4.5-fold) and triggering cytotoxicity receptor NKG2D (by 6.4-fold), which might account for the enhanced cytotoxicity of NK cells [59].

A very extensive and well-documented report concerning the biphasic effects induced by RES was published by Posadino et al. [60]. The authors investigated numerous in vitro endpoints in HUVEC incubated with increasing concentrations of RES and undertook an ambitious attempt to elucidate the mechanism of the observed processes. It was found that at 1 μM RES intracellular basal level of ROS was decreased by 35% whereas higher concentrations (10 and 50 μM) enhanced the ROS level by 25% and 50%. Cell viability was slightly insignificantly (15%) increased when exposed to 1 μM of RES. Higher concentrations (10 and 50 μM) caused a significant decrease in cell viability, 40% and 60%, respectively. Consistently this pattern of results was reflected in the assay for DNA synthesis. The lowest RES concentration increased DNA synthesis by 15%; higher concentrations suppressed this parameter by 40% and 80%. The expression of antiapoptotic gene Bcl-2 in HUVECs treated with RES also followed biphasic concentration-response mode. At 1 μM RES increased Bcl-2 mRNA levels by 48%. The effects of higher RES concentration were the opposite—an expression of this gene was significantly diminished by 54% and 86%. These findings confirmed that RES at high concentration induced apoptosis in HUVECs. Similarly, the expression of two other genes playing an essential role in cell cycle progression and cell proliferation, namely c-myc and ornithine decarboxylase (ODC), displayed a biphasic response to RES. A higher RES concentration significantly decreased the mRNA levels of both genes by 30–43% whereas their expression was enhanced in cells treated with 1 μM RES by 27% and 47%. It was also demonstrated that RES biphasically modulated protein kinase C (PKC) activity in HUVECs. The lowest concentrations caused a 2.1-fold increase in PKC activity, whereas higher concentrations exerted a strong inhibitory effect by 56% and 72%, which was consistent with the biphasic effect of RES on ROS production [60]. The above findings contribute significantly to the understanding of the mechanism of RES concentration-dependent effects.

The only in vivo research concerning the biphasic effects of RES was reported by Juhasz et al. [61]. The biphasic cardioprotective effect was demonstrated in rats fed 3 doses of RES for 30 days. Their hearts were isolated and subjected to ischemia/reperfusion. The lowest dose, 2.5 mg/kg conferred maximum protection as evidenced by a 50% increase in aortic flow and left ventricular developed pressure, as well as infarct size, decreased by 40%. At 25 mg/kg cardiac function parameters were significantly reduced; at 100 mg/kg no aortic flow and no developed pressure were detected, indicating that the heart did not function. The authors suggested that this protective effect of RES was exerted through its ability to induce gentle intracellular stress, leading to the upregulation of the defense system. At high doses RES depressed cardiac function and induced apoptosis, which is in agreement with the well-known properties of RES concerning the inhibition of RNA, DNA and protein expression, chromosomal aberration and the inhibition of cell proliferation [2].

The reports presented in this section confirm the previous findings [2,3] that low concentrations of RES (1–100 µM) stimulate the proliferation of various cell lines, whereas higher concentrations (50–1000 µM) inhibit cell viability. The difference in magnitude of concentrations stimulating or inhibiting DNA synthesis was not so distinct, 1–10 µM vs. >15 µM, respectively. Much lower concentrations (0.1–1 µM) were able to protect DNA which has been shown by decreased chromosomal instability (CIN), whereas 100 µM of RES increased this parameter.

The increase in proliferation was explained by the enhanced expression of cellular defense genes resulting from mild oxidative stress as well as by activating ERKs and p38 kinases. Prooxidant properties of RES contributed to its antiproliferative action demonstrated at higher concentrations, as evidenced by NADP oxidase activation, superoxide anion generation and an increase in ROS level. Other beneficial effects of RES low concentrations presented here include a decreased stem cell senescence, antigenotoxic effect, enhanced myoblast plasticity and antiapoptotic action.

Summing up, at higher doses/concentrations, RES can act as a preventive agent with respect to carcinogenesis, the opposite effect of low concentration suggests a need for caution [2].

Data on biphasic concentration-response induced by RES are summarized in Table 2.

## 4. Other Phytochemicals

The isothiocyanate **sulforaphane** (SFN) found in high concentrations in cruciferous vegetables has gained extensive research interest due to its anticancer and chemopreventive properties [62,63]. SFN is considered to be a hormetic molecule [1,64,65]; however, a thorough literature search revealed that very few articles are available in which a specific biphasic dose-response relationship is reported, and only these reports were selected to be presented in the current review.

Bao et al. [63] presented a study on biphasic dose-response promoted by SFN in a high number of cultured cells demonstrating that a low concentration of SFN (1–5 μM) stimulated cell growth by 20–40% as compared with controls, whereas a high concentration (10–40 μM) inhibited cell growth in some tumor cell lines: bladder cancer T24, hepatoma HepG2, and colon cancer Caco-2. A similar dose-response relationship was observed in regular cell lines, including hepatocytes HHL-5, colon epithelial CCD841 cells, and skin fibroblasts CCD-1092 SK. The migration of T24 cells also followed the biphasic dose-response manner. Incubation with 2.5 and 3.75 μM SFN increased this parameter to 128% and 133% of the corresponding controls. Concentrations higher than 5 μM decreased cell migration, which was ceased at 40 μM. A low concentration of SFN (2.5–5 μM) promoted tube formation (a marker of angiogenesis) by 18% as evidenced by 3D angiogenesis assay. Concentrations 10 and 20 μM inhibited tube formation decreasing it to 61% and 20% of the control. The authors suggested that the mechanism of cell growth stimulation by low SFN concentrations may be related to the activation of growth-promoting molecules (for example RAS, RAF, ERK, PI3K) and signal transduction pathways such as NF-kB, FOXO, Nrf2 [63].

The concentration of SFN in human plasma after consumption of cruciferous vegetables can reach 1–5 μM, the level which promotes cell growth. The authors suggested that it might explain some inconsistency of epidemiological findings regarding the association between isothiocyanates intake and cancer risk [63].

Biphasic effects of SFN were also demonstrated in human mesenchymal stem cells (MSCs). A low concentration of SFN (0.25 and 1 μM) stimulated proliferation of MSCs by 22%, whereas 20 μM caused a significant, about 60% reduction of cell growth. Similarly, the concentration of SFN up to 5 μM reduced the number of apoptotic cells with a maximum effect of 76% demonstrated by 0.25 μM. On the contrary, concentration 20 μM caused a 2.3-fold increase in the percentage of apoptotic cells. The number of senescent cells—as assessed by acid-β-galactosidase assay—was decreased by about 30% in MSCs incubated with 0.25 and 1 μM SFN. High doses of SFN (5 and 20 μM) increased senescent cell number by 62% and 4-fold, respectively. The production of cellular ROS was also affected by SFN in a biphasic manner. Low concentration (0.25 μM) reduced by 30% the production of ROS in the basal state and under stress condition. The concentration of 20 μM caused a 30% increase in ROS generation. The authors suggest that SFN should be used as an anticancer agent very carefully because the compound may impair healthy stem cells that support hematopoiesis and contribute to homeostatic maintenance [66].

A stimulating effect of a low concentration of SFN, up to 5 μM on cell proliferation was demonstrated using various cell lines: 16% increase in MCF-7 [67], 10% in HHL-5 (human hepatocytes) [68], 16% in HepG2 cells [68] as well as 30% increase in human lymphoblastoma cells [69]. At a concentration higher than 5 μM, the proliferation of every cell line was substantially inhibited as compared to controls. Misiewicz et al. [69] additionally investigated intracellular glutathione content and revealed that incubation of lymphoblastoma cells with 0.5–5 μM SFN caused a 39–340% increase in this parameter, however, the concentration 10 μM decreased the GSH level to 50% of control value.

The biphasic effect of **berberine** (BER), an isoquinoline alkaloid, on the cell growth was demonstrated in five cancer cell lines: murine melanoma cell line B16-F10, human breast cancer cells MDA-MB-231, MDA-MB-468 and MCF-7, and human colon cancer cells LS-174. At low concentrations (1.25–5.0 μM) berberine stimulated the growth of all types of cells by 12–70% as compared to controls. Higher concentrations of BER (10–80 μM) inhibited cell proliferation up to 90% [70].

Consistent with these findings, co-treatment with a low dose of BER significantly attenuated the anticancer activity of chemotherapeutic drugs: fluorouracil, camptothecin, and paclitaxel. The authors suggested that BER activates the protective stress response in cancer cells as evidenced by the up-regulation of MAPK/ERK1/2 and PI3K/AKT signaling pathways, which can partly explain the observed effects [70].

Berberine also exerted biphasic dose-response effect on the viability of another type of cells, phaeochromocytoma cell line PC-12. A low concentration of BER (0.1–1.0 μM) significantly increased the viability of PC-12 cells, maximum by 40%, whereas 2–64 μM of BER inhibited cell viability, decreasing it to 50% of the control value [71]. Additionally, on the basis of several assays, the authors suggested that BER protects against 6-hydroxydopamine (6-OHDA)-induced neurotoxicity in PC12 cells through the hormetic mechanism. Low concentrations of BER (0.25–1.0 μM) protected cells from 6-OHDA-induced cytotoxicity and apoptosis, higher concentrations (2–16 μM) did not show this effect. The authors speculated that PI3K/AKT/Bcl-2 pathway was involved in protective effect of low BER concentration. In zebrafish larvae, low doses of BER (0.3–1.3 μM) alleviated the loss of dopamine neurons caused by 6-OHDA treatment, no protective effect of a high dose of BER (20 μM) was observed. The same range of BER low doses reversed the 6-OHDA-induced reduction of larvae locomotor activity, whereas the high dose effect was very slight [71]. In all experiments referring to the neuroprotective activity of BER, no biphasic dose-response was shown since the high dose of BER did not exert an effect opposite to that observed for low doses. Thus, the objection arises whether these relationships can be considered hormetic.

The effect of the pretreatment with two polyacetylenes, **falcarinol** and **falcarindiol** on cellular stress in primary myotube cultures exposed to hydrogen peroxide was investigated. At a lower concentration of both compounds (1.6–25 μM) the formation of ROS was slightly enhanced (maximum by 10–30%). Parallelly an increase in glutathione peroxidase (GPx) mRNA expression, as well as a decreased Hsp70 and heme oxygenase1 (HO-1) mRNAs, was observed. Preincubation with higher concentrations of the compounds tested, 50 and 100 μM resulted in a substantial decrease in ROS formation (to about 10% of the control value) and GPx mRNA expression as well as the increased expression of mRNA for HSP70 and HO-1. Myoblast viability was also affected by falcarindiol in a biphasic manner. The lower concentrations of the compound (0.61–9.8 nM) increased the viability of myotubes slightly (19%). Higher concentrations (2.5–5 μM) suppressed the viability significantly, by about 96%. The authors suggested that a protective effect of both polyacetylenes was associated with the induction of antioxidant enzyme, GPx [72].

The biphasic effect of falcarinol was also demonstrated in another experiment in which the proliferation of primary bovine mammary epithelial cells was measured using the bioassay based on the incorporation of tritiated thymidine into cellular DNA. Falcarinol exerted stimulatory effects (maximum 26%) at concentration ~0.04–0.20 μM and inhibited cell growth between ~4 μM and ~41 μM with the maximum effect (90%) observed at ~41 μM [73].

Young et al. [74] reported on the biphasic effect of falcarinol on the proliferation of the human colon carcinoma cell line CaCo-2. The increase in cell proliferation was observed at the concentration range 1–10 μM, with 1 μM being the most effective (80% increase). At concentrations above 20 μM proliferation of cells decreased gradually to reach 15% of the control value. Concomitantly the expression of apoptosis indicator, caspase-3, and basal DNA strand breakage was decreased at a low concentration of falcarinol by 50% and 40%, respectively. At concentrations above 20 μM a 13-fold enhancement of caspase-3 expression, as well as a 2-fold increase in DNA strand breakage, were observed [74].

Chattopadhyay et al. [75,76] investigated the effects of two flavonoids on longevity in *Drosophila melanogaster*. **Rutin** (quercetin-3-rutinoside) was shown to extend the median lifespan in female flies at a concentration of 200 and 400 μM by 30% and 43%, respectively. The treatment of flies with higher concentrations, 600 and 800 μM resulted in a decrease in survival, by 13% and 16%. The transcript levels of genes associated with longevity were increased in flies treated with lower doses of the compound [76]. In another experiment, *D. melanogaster* was fed a diet containing **naringenin** (4′,5,7- trihydroxyflavanone) at a concentration of 50–800 μM. Concentrations 200 and 400 μM caused an increase in the lifespan of male and female flies by 13% and 23%. Administration of higher doses, 600 and 800 μM, resulted in a decrease in lifespan by 14% and 30%, respectively. A standard diet supplemented with 200 μM naringenin increased the percentage of pupae formation as well as the number of flies that eclosed after pupation, whereas the sharp decline of both endpoints was observed when the content of naringenin was 600 and 800 μM [75].

**Luteolin** (3′,4′,5,7-tetrahydroxyflavone) was shown to increase the viability of MCF-7 cells at concentrations 1–10 μM by about 18%. Higher concentrations of the compound, 30–1000 μM caused a decrease in cell viability to about 95% of the control value [77]. The biphasic effect of luteolin on autophagy was demonstrated in HepG2 cells. At concentrations up to 35 μM luteolin caused about a 45% increase in the level of LC3-II, a marker of autophagy. Higher concentration (~105 μM) decreased this parameter by 35%. Autophagy is an essential process for cell homeostasis, and its impairment contributes to the pathogenesis of various diseases [78]. The antimutagenic activity of some flavonoids of rooibos (*Aspalathus linearis*) displayed a biphasic dose-response relationship. *Salmonella typhimurium* mutagenicity assay was used with 2-acetamido-fluorene (2-AAF) and aflatoxin B1 (AFB1) as model mutagens. **(+) Catechin** and **rutin** displayed a co-mutagenic effect at concentrations 1.2 and 0.8 mM, respectively, and antimutagenic activity at lower concentrations (0.01–0.6 mM) in a 2-AAF assay. On the contrary, **luteolin** was co-mutagenic at the lowest concentration tested (0.006 mM) and antimutagenic at higher concentration (1.2 mM) in the same assay [35].

The rat PC12 cell line was pretreated with **Z-ligustilide**, a bioactive phthalide isolated from Rhizoma Chuanxiong. Then cells were subjected to oxygen-glucose deprivation (OGD) procedure. At a low concentration (1–25 μM) Z-ligustilide protected cells from OGD-induced apoptosis and increased cell viability by about 50%. The protective effect of the compound declined with increasing concentrations to 73% of the basal level at 50 μM. The authors suggested that low concentrations of Z-ligustilide triggered moderate ROS production in cells which stimulated the cellular defense system via activation of PI3K/AKT and Nrf2/HO-1 pathways [79]. Yi et al. [80] reported on the biphasic effects of Z-ligustilide on selected enzymes’ activity in *Spodoptera litura* larvae. Low doses of the compound (0.1–0.5 mg/g diet) increased the activities of glutathione S-transferase (GST) (by 23%), cytochrome P450 (by 150%), acetylcholinesterase (by 123%) and carboxylesterase (by 50%). Doses 1 mg/g and 5 mg/g decreased the activity of these enzymes by 80–97% except for carboxylesterase. A similar biphasic dose-response relationship was observed for mRNA expression of GSTS1, CYP4S9, and CYP 4M14. The authors suggested that a low dose of Z-ligustilide stimulated Nrf2 mediated detoxification enzymes and HSP70 pathways [80].

**Salvianolic acid B** (a condensate of three molecules of danshennol and one molecule of caffeic acid) exhibited a biphasic effect on the total metabolic activity of a rat mesenchymal bone marrow cell culture. Low concentrations, ~4–111 μM, of the compound tested increased the metabolic activity by 40%, whereas ~223 μM caused almost complete inhibition. A similar type of effect was found with the ALP activity. Lower concentrations of salvianolic acid increased the enzyme activity by 40%. The highest concentration tested entirely suppressed ALP activity. As ALP is an indicator of early osteoblast differentiation, the authors concluded that salvianolic acid has the potential to ameliorate bone healing [81].

**Glyceollin I** a compound classified as prenylated pterocarpan (an induced phytoalexin isolated from soybean) demonstrated a biphasic effect on yeast life span. At low concentration (10–100 nM) glyceollin I induced a chronologic life span (CLS) extension with the maximum effect 40%, relative to the control. A concentration higher than 1.0 µM led to the reduction of CLS and toxicity [82].

**Umbelliprenin**, a natural sesquiterpene coumarin, affected apoptosis in Jurkat T-CLL cells in a biphasic fashion. Concentration 10 µM and 25 µM increased apoptosis by about 20%, whereas the concentration 50 and 100 µM decreased apoptosis by 50% below the level observed in control cells [83].

**Nantenine**, an aporphine alkaloid isolated from *Ocotea macrophylla*, affected the activity of K+ -p- nitrophenylphosphatase (K+ -p-NPPase) in synaptosomal membranes isolated from rat brain in a biphasic manner. Concentrations 50 and 0.3 mM increased the activity of the enzyme by about 20% and 40%, respectively. Concentrations higher than 0.75 mM suppressed the activity almost entirely. These findings might explain the previously observed different effects of nantenine on seizures. The authors suggested that the anticonvulsant action of nantenine is attributed to the stimulation of K+ -p-NPPase activity by low doses of alkaloid. The convulsant effect of the compound at high doses might be related to the enzyme inhibition [84].

Kafi et al. [85] demonstrated a biphasic effect of a lignan compound, **arctigenin** on the expression of antiapoptotic gene Mcl-1 in the K562 leukemia cell line. At concentrations, ~27 and ~54 μM arctigenin increased the gene expression by 75%. Concentrations 2-fold greater caused a 75% decrease in the gene expression [85].

Hunt et al. [86] reported that two naphthoquinone compounds, **plumbagin** and **naphazarin** extended the lifespan of *Caenorhabditis elegans* by 10% and 17% when nematodes were exposed to their lower concentrations (1–45 μM plumbagin and 50–500 μM naphazarin). Higher concentrations of plumbagin and naphazarin, 100 and 1000 µM, caused about 90% and only 9% reduction of a lifespan, respectively. The authors found that CNC transcription factor, SKN-1, which promotes antioxidant gene expression, mediates a beneficial effect of both compounds at low concentrations [86].

**Rosmarinic acid** (RA) [caffeic acid ester of 3-(3,4-dihydroxyphenyl) acetic acid] was shown to affect the lifespan in *C. elegans* in a biphasic manner. At concentrations 100–300 µM the lifespan was extended by 10% at 200 µM, whereas the treatment with concentration 600 µM resulted in a 6% decrease in lifespan. The increased expression of six hsp genes was determined in nematodes treated with RA, which suggested the involvement of stress response activation in the observed effect [34].

A similar experimental model was used to examine the biphasic effects of **epigallocatechin-3- gallate (EGCG)**. Treatment of *C. elegans* with EGCG in the concentration range of 50–300µM resulted in increased longevity (by 5–16%). Higher concentrations of EGCG (800–1000 µM) shortened lifespan by 8% and 14%, respectively. The authors suggested that the life-extending mechanism was stimulated by EGCG-induced ROS production and involved an inducible AMPK/SIRT1/FOXO-dependent redox signaling pathway [87].

The biphasic effects of **panaxatriol saponins** (PTS) isolated from *Panax notoginseng* were examined in PC-12 cells. A stimulatory effect on cell proliferation was observed at concentrations 0.03–1.0 mg/mL and peaked at 0.12 mg/mL (30% increase). The concentration of 4 mg/mL very slightly by 10% reduced cell proliferation. A similar pattern of results was gained in PC12 cells with 6-OHDA induced damage. At low concentrations (0.03–2.0 mg/mL) PTS increased cell viability by 24%. However, co-treatment with a higher concentration of PTS (4 mg/mL) resulted in further inhibition of cell growth by 16%. The authors postulated that PTS exerted neuroprotection against 6-OHDA-induced cell damage in PC-12 cells through activating the PI3K/AKT/mTOR cell proliferation pathway and AMPK/SIRT1/FOXO3 cell survival pathway. They also pointed out the potential application of PTS for the prevention and treatment of neurodegenerative diseases [88]. The biphasic effect of PTS was not confirmed in the zebrafish larvae model. Concentrations 0.01–0.1 mg/mL reversed the dopamine neuron loss induced by 6-OHDA. The higher concentration of PTS (10 mg/mL) neither exerted protection against neuron loss nor caused the opposite effect [88].

The effect of increasing concentrations of **cynarin** (1,3-O-dicaffeoylquinic acid) (CYN) found in artichoke, on cell proliferation was tested in normal human skin fibroblasts (FSF-1) and telomerase-immortalized mesenchymal stem cells (hTERT-MSC). Both cell lines showed biphasic concentration-response to CYN. Concentrations 1–50 µM caused a 10–26% increase in the number of FSF-1 cells, whereas higher concentrations (75–500 µM) decreased cell survival by 16–84%. Similarly, lower CYN concentrations, 1–10 µM, increased the survival of hTERT-MSC cells by 7–60%, and higher concentrations inhibited cell growth by 10–96%. The authors suggested that the increase in cell growth might be due, in part, to the induction of stress response by lower CYN concentrations, as evidenced by an increase in the expression of heme oxidase-1 [89].

The dose-response relationship for the carcinogenic effect of **caffeic acid** (CA) was investigated in male F344 rats fed for 4 weeks a diet containing different CA concentrations: 0.05%, 0.14%, 0.40%, and 1.64% treatment [90]. In the forestomach, a target organ of CA-induced carcinogenesis, the markers of cell proliferation, the total number of epithelial cells, and the number of S-phase cells, were increased about 2.5-fold at 0.40% and 1.64%. At 0.14% both variables were decreased by about 30%. The authors suggested that this low-dose effect could explain the well-known cancer-protective properties of caffeic acid. The lowest dietary concentration tested in the experiment was equivalent to 35 mg/kg b.w./day. This dose is much lower than that enhancing cell proliferation in the rat forestomach and lower than ingested by strong coffee drinkers. Hence, it is in the range of potential protection, assuming the extrapolation of these outcomes to humans [90].

Data on biphasic concentration/dose-dependent effects discussed in this section are collected in Table 3.

## 5. Comments

Apparently, there are a lot of reviews concerning biphasic dose/concentration-response to phytochemicals. However, critical analysis of their content reveals that some of them refer mainly to numerous aspects of beneficial health effects and underlying mechanisms, and no single reference related to biphasic dose-response is cited, for example [91,92,93,94]. The common feature of this kind of articles is that some phytochemicals are demarked “hormetic” solely on the basis of the induction of “adaptive stress response” or “cellular defense system” at low doses. These effects are counteracted a priori with the presumed toxicity of high doses. In our opinion, such interpretation is not justified because the opposite effects of high doses on endpoints tested were not experimentally evidenced.

The current review includes only original reports on experiments which results conform to the classic definition of biphasic hormetic like dose-response.

The majority of studies presented here were performed on cell cultures. The most common endpoint tested was a proliferation of tumor and non-cancerous cells. Therefore, the question arises: why for other endpoints this pattern of dose-response has been reported rather rarely? Is it due to the fact that such type of response is limited to simple parameters, or maybe other endpoints were not examined with respect to biphasic dose-response? This issue should be addressed in future research.

**Figure 2:**
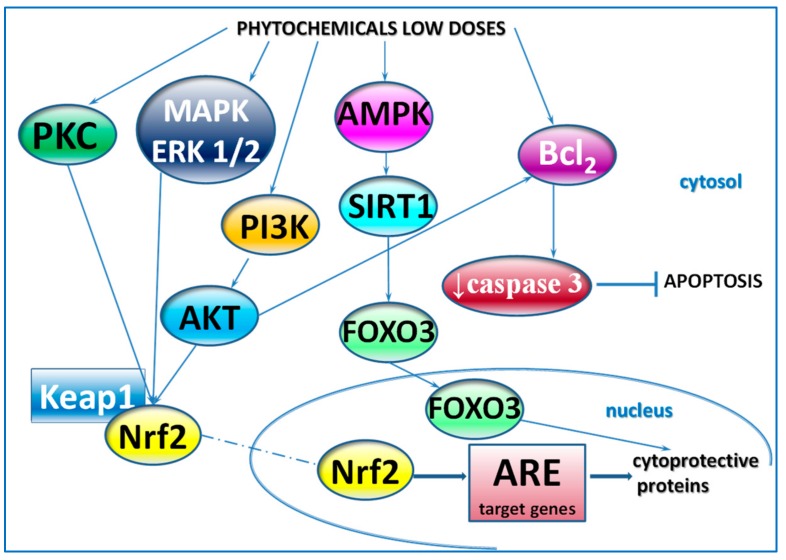
Examples of mechanisms involved in the cellular response to low doses of phytochemicals–on the basis of findings presented in the current review. Phytochemicals can activate kinase cascades, including PKC, MAPK/ERK1/2, PI3K/AKT, which play a critical role in the regulation of cell growth, proliferation, survival, and apoptosis. Downstream effector of these kinases is transcription factor Nrf2, which is released from the complex with keap1 and translocates to the nucleus, binds to ARE and stimulates the expression of cytoprotective proteins, e.g., antioxidant enzymes and phase-2 proteins. SIRT-1 plays a key role in the cellular response to various stressors by activating transcription factor FOXO3, which induces genes encoding cytoprotective proteins. The transcriptional activity of FOXO3 is modulated by both AMPK and SIRT-1. PI3K/AKT is the major pathway mediating cell survival and inhibiting apoptosis. Bcl-2, a pro-survival, anti-apoptotic, and cytoprotective molecule, can be activated directly by chemicals or via PI3K/AKT pathway. AKT—serine/threonine protein kinase; AMPK—AMP-activated protein kinase; ARE—antioxidant response elements; Bcl2—B-cell lymphoma 2; ERK—extracellular signal-regulated kinase protein-serine/threonine kinase; FOXO3—forkhead box O3; KEAP1—Kelch-like ECH-associated protein 1; MAPK—mitogen-activated protein kinase; Nrf2—nuclear factor erythroid 2-related factor 2; PI3K—phosphatidylinositol 3-kinase; PKC—protein kinase C; SIRT1—sirtuin1.

The overwhelming part of the reports presented in the current review did not contain the elucidation of the mechanism of the biphasic response to phytochemicals. Proliferative activity of low phytoestrogens concentrations was generally explained on the basis of transactivation of the estrogen receptor [9,10,11,12,13,14,15,16,17,18,19,21,22,23,29,37,38,39,40,44]. In more recent articles, some molecular aspects involving the induction of genes expression or activation by phytochemicals of various signaling pathways, for example, MAPK/ERK1/2 and PI3K/AKT were revealed [48,49,57,60,63,72,74,78,79]. In the *Caenorhabditis elegans* model, the increased expression of some genes involved in lifespan or stress response was associated with extended lifespan [34,86,87]. Examples of mechanisms involved in the cellular response to low doses of phytochemicals are presented in Figure 2.

The current review supports the opinion of many authors that the stimulatory effects of low doses/concentrations are not always beneficial [8,95] as evidenced by the increased proliferation of tumor cells exposed to phytochemicals. On the other hand, the enhanced proliferation of neuron-like PC-12 cells induced by some phytochemicals accounts for their neuroprotective action [71,88]. Moreover, the interpretation of the impact of a stimulatory effect depends on the context of a potentially therapeutic application. Chirumbolo et al. reported that low concentrations of quercetin enhanced activation of basophils [32] and simultaneously caused an increase in histamine release [33]. The first effect was considered beneficial for the strengthening of an inflammatory reaction against invading bacteria, but the latter was harmful in the context of the potential use of quercetin in the prevention of allergy.

**Figure 3:**
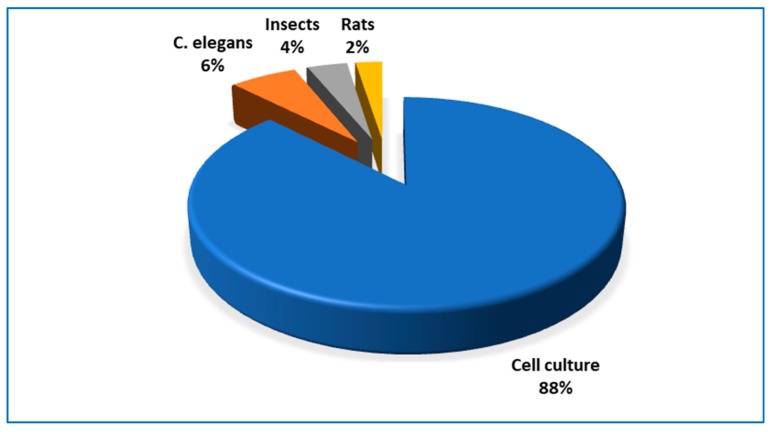
The percentage share of various types of experimental models applied in the reports cited in this review.

It is intriguing how few experiments referring to biphasic dose-response induced by phytochemicals were carried on animal models, as demonstrated in the current review. In *C. elegans* [34,86,87] and *D. melanogaster* [75,76] treated with the compounds tested, biphasic changes of lifespan were recorded. Selected enzymes’ activity in *Spodoptera litura* larvae [80] and mutagenic activity tested by *Salmonella* *typhimurium* assay were modulated in a biphasic manner [35,36]. In transgenic mouse models [20,88] as well as in the zebrafish larvae model [71,88] solely the effects of low doses of compounds tested were demonstrated to be consistent with in vitro findings, however, no opposite effects of high doses were recorded. We found only two experiments on rodents in which regular biphasic dose-response was shown. One referred to the changes in markers of cell proliferation in the forestomach of rats fed a diet containing various amounts of caffeic acid [90], another described the cardioprotective effect of resveratrol administered to rats for 3 months [61] (Figure 3).

Data on the biphasic dose-response of various endpoints to phytochemicals may have a potential therapeutic or preventive implication. However, their significance is compromised by the fact that very few in vitro findings were supported by in vivo experiments. Therefore, the feasibility of extrapolating results from cell culture models to the whole organism might be questioned. The fact that low concentrations of some phytochemicals can stimulate proliferation should raise concerns with regard to carcinogenesis. However, concentrations tested in cell cultures may not be relevant to the whole organism and in various organs, different doses can evoke different effects [96]. For better extrapolation from in vitro biphasic dose-response data to in vivo conditions physiologically based pharmacokinetic models (PBPM) should be used taking into account expected plasma and tissue concentration as well as processes of biotransformation [28]. The need for caution in the assessment of pharmacological effect is supported by the report by Lutz et al. [90]. Conversely to the majority of data presented in the current review, the authors showed that high doses of caffeic acid displayed proliferative effects, whereas low doses decreased cell division in the forestomach of rats [90].

Some authors argue that biphasic dose-response is affected by a lot of factors rendering the adequate assessment of potential health benefit impossible. This type of dose-response can be differential among endpoints in a given system/model i.e., some endpoints may demonstrate positive or negative effects whereas some others may be unresponsive or clinically insignificant [97]. Moreover, low and high doses are not unequivocally defined because the low doses used in the in vitro experiments might be high doses if extrapolated to the whole organism [96].

Evidence for adverse effects of phytochemicals, depending on their concentrations/doses should provoke further mechanistic investigations to elucidate the phenomenon of their biphasic/hormetic action. Given the essential role of plant-based food in human nutrition, further preclinical and human studies aiming at establishing a safe and efficient dose of phytochemicals are required.

## Figures and Tables

**Table 1:** Phytoestrogens displaying biphasic concentration-response relationship.

Compound *	Model	Concentration	Effects	Mechanism	Refs
	**Effects Linked to Estrogenic Activity**				
Artelastin Artelastocarpin Artelastochromen Carpelastofuran isolated from *Artocarpus elasticus*	MCF-7	0.02–2.90 μM	↑proliferation, DNA synthesis		[38,39]
		>3.12 μM	↓proliferation		
		25 μM	↓DNA synthesis		
Biochanin A	MCF-7	~4–35 μM	↑proliferation		[37]
		~106–352 μM	↓proliferation		
		~18 μM	↑DNA synthesis		
		~70 μM	↓DNA synthesis		
	T-47D	~4 μM	↑proliferation	↓p53	[24]
		~70 μM	↓proliferation	↑p53	
Breviflavone B isolated from *Epimedium brevicornum*	MCF-7	450 nM	↑proliferation		[40]
		2.2–6.6 μM	↓proliferation	↓ERα	
Daidzein	T-47D	~1–79 μM	↑proliferation	↓p53	[24]
		~157 μM	↓proliferation	↑p53	
	MCF-7	~1 μM	↑proliferation		[25]
		>10 μM	↓proliferation		
	LoVo	0.1, 1.0 μM	↑proliferation		[26]
		10–100 μM	↓proliferation	G0/G1 arrest	
				↑caspase-3	
	KS483, mouse bone marrow cells	<20 μM	↑osteogenesis ↓adipogenesis	PPARs transactivation	[27]
		>30 μM	↓osteogenesis ↑adipogenesis		
Enterodiol Enterolactone	MG-63	~33 μM	↑viability	↑*osteonectin* ↑*collagen I*	[43]
		~33–333 μM	↑ALP activity		
		>333 μM	↓viability	↓*osteonectin* ↓*collagen I*	
		~3–33 mM	↓ALP activity		
Genistein	MCF-7	<1 μM	↑proliferation	↑ER transcription	[11,12,13,14,15,17,18,19]
		>10 μM	↓proliferation		
	PC-3	500–1000 nM	↑proliferation,	↑MMP-9 activity	[20]
				↑osteopontin	
		50,000 nM	↓proliferation	↓MMP-9 activity	
	RWPE-1	1.5–12.5 μM	↑proliferation	↑ERK1/2 activity	[21]
		50 and 100 μM	↓proliferation		
	UtLM	~4 μM	↑proliferation		[22]
			↑PCNA, ↑cells in S phase		
		>37 μM	↓proliferation ↑apoptosis		
	KS483, mouse bone marrow cells	0.1–10.0 μM	↑osteogenesis ↑ALP activity		[23]
			↑nodule formation and calcium deposition		
		>25 μM	↓osteogenesis ↓ALP activity		
			↓nodule formation and calcium deposition		
	KS483, mouse bone marrow cells	0.1–1.0 μM	↓adipocytes number		[23]
		10–50 μM	↓adipocytes number		
Glabrene isolated from *Glycyrrhiza glabra*	T47-D, MCF-7	100 nM–10 μM	↑proliferation		[42]
		>15 μM	↓proliferation		
Glabridin isolated from *Glycyrrhiza glabra*	T-46D	0.1–10 μM	↑proliferation		[41]
		>15 μM	↓proliferation		
Isoliquiritigenin synthesized by authors	MCF-7	<1 μM	↑proliferation		[44]
		10 μM	↓proliferation		
Kaempherol	MCF-7	<1 μM	↑proliferation		[46]
		>1 μM	↓proliferation		
Quercetin	MCF-7	<1 μM	↑proliferation		[17]
		>10 μM	↓proliferation		
	HCT-116	1–30 μM	↑proliferation		[28]
		40–100 μM	↓proliferation		
	HT-29	1–67 μM	↑proliferation		
		80–100 μM	↓proliferation		
	SCC-25	1–10 μM	↑proliferation		[29]
		>100 μM	↓proliferation		
	**Activity not Linked to Estrogenic Properties**				
Isoliquiritigenin	HUVEC/PMA	<10 μM	↑TIMP-2	↓JNK, p38 MAPK pathway	[45]
		25 μM	↓TIMP-2		
Quercetin	RAW 264.7	10–100 nM	↑PGE2		[30]
		10–100 μM	↓PGE2		
	basophils/fMLP	~0.03–0.33 μM	↑CD63, CD203c		[32]
		~3–33 μM	↓CD63, CD203c		
	basophils/fMLP	0.03–0.3 μM	↑histamine	PI3K involvement	[33]
		33 μM	↓histamine		
	*Caenorhabditis elegans*	100–200 μM	↑lifespan	↑*hsp*	[34]
		250 μM	↓lifespan		
	*Salmonella typhimurium*/AFB1	0.006–0.01 mM	↓mutagenicity		[35]
		0.06–0.12 mM	↑mutagenicity		
	*Salmonella typhimurium*/MeIQ	0.1, 1 μM	↑mutagenicity, CYP1A2 activity		[36]
		50, 100 μM	↓mutagenicity, CYP1A2 activity		

* If the source of the compound was not specified it was obtained commercially; ↑ = increase, ↓ = decrease; 2-AAF—2-Acetylaminofluorene; AFB1—aflatoxin B1; ALP—alkaline phosphatase; CD203c—basophil-specific ectoenzyme E-NPP3; CD63—tetraspan transmembrane protein family; CYP1A2—Cytochrome P450 1A2; fMLP—bacterial formyl peptide N-formylmethionine-leucine-phenylalanine; HCT-116, HT-29—colon carcinoma cell line; HepG-2—human liver cancer cell line; HUVEC—human umbilical vein endothelial cell line; KS483—murine osteoprogenitor cell line; JNK—c-JUN terminal kinase; LC3-II—microtubule-associated protein 2 light chain 3; LoVo—human colon adenocarcinoma cell line; MCF-7—human breast adenocarcinoma cell line; MeIQ—2-amino-3, 4-dimethylimidazo [4,5-f]quinoline; MG-63—human osteoblast-like cells; MMP-9—matrix metallopeptidase 9; p53—tumor protein p53; PC-3—human prostatic carcinoma cell line; PCNA—proliferating cell nuclear antigen; PGE2—prostaglandin E2; PI3K—phosphoinositide-3 kinase; PMA—phorbol myristate acetate; RAW 264.7—murine macrophage cell line; p38 MAPK—p38 mitogen-activated protein kinase; SCC-25—oral squamous carcinoma cell line; T-47D—human breast cancer cell lines; TIMP-2—tissue inhibitor of metalloproteinase-2; UtLM—human uterine leiomyoma.

**Table 2:** Biphasic concentration/dose-response relationship induced by resveratrol.

Model	Concentration	Effects	Mechanism	Refs
NHEK	<50 μM	↑viability	↑CAT, Nrf2, KEAP1, NQO1, GCLC, GSR, G6PD, FOXO3, SIRT1, DAPK 1 (5–100 µM)	[48]
	500 μM	↓viability	↓CAT, Nrf2, KEAP1, NQO1, GCLC, GSR, G6PD, FOXO3, SIRT1, DAPK1 150 µM	
NHDF	1–300 μM	↑viability		
	500 μM	↓viability		
HepG2	1–100 μM	↑viability		
	500 μM	↓viability		
NPCs	1, 10, 20 μM	↑proliferation	↑ERK1/2, p38, p-CREB, Bcl-2, TrkA, synaptophysin, PSA-NCAM	[49]
	50, 100 μM	↓proliferation	↓p-ERK1/2, p-p38 MAPK	
			↑caspase-3	
HT-29	1–10 μM	↑proliferation		[50]
	50, 100 μM	↓proliferation	↑NADPH oxidase activity, ↑ɣH2AX, SIRT6	
Bovine spermatozoa	1–50 μM	↑viability		[51]
		↓superoxide anion production		
	100, 1000 μM	↓viability		
	100, 200 μM	↑superoxide anion production		
LNCaP	5 μM, 10 μM	↑DNA synthesis	↓p21cip1, p27kip1	[52]
			↑Cdk2 activity	
			↑cyclins A, E	
	>15 μM	↓DNA synthesis		
Rat ovarian	10 μM	↑DNA synthesis		[53]
granulosa cells	30,50 μM	↓DNA synthesis		
Normal colon epithelial cells	0.1–1 μM	↓chromosomal instability, ↑viability	↑*SAC*	[54]
	100 μM	↑chromosomal instability, ↓viability	↓*SAC*	
C12C12	10 μM	↑cell motility		[55]
	40–60 μM	↓cell motility	↓miosin Tpe1 and total ATPase activity	
HepG2	1, 10 μM	↑apoM,		[56]
	100 μM	↓apoM		
hMSCs	0.1 μM	↓cellular senescence	↑*Sirtuin1*	[57]
	5, 10 μM	↑cellular senescence	↓*Sirtuin1, Sirtuin2, Birc4, Birc5*	
			↑*Cdk2*	
HepG2/4NQO	10, 25, 50 μM	↓genotoxicity		[58]
	100, 250 μM	↑genotoxicity		
NK	1.56, 3.13 μM	↑cytotoxicity	↑*NKG2D, NKG2D*	[59]
			↑*IFN-γ, IFN-γ*	
	25, 50 μM	↓cytotoxicity		
HUVEC	1 μM	↓ROS	↑*Bcl-2, c-myc, ODC*	[60]
		↑viability, DNA synthesis	↑PKC activity	
	10, 50 μM	↑ROS	↓*Bcl-2, c-myc, ODC*	
		↓viability, DNA synthesis	↓PKC activity	
Rats	2.5 mg/kg	↑aortic flow, LVDP, ↓infarct size	↓cardiomyocyte apoptosis	[61]
	25 mg/kg	↓aortic flow, LVDP, ↑infarct size	↑cardiomyocyte apoptosis	
	100 mg/kg	no heart function	↑cardiomyocyte apoptosis	

↑ = increase, ↓ = decrease; 4NQO—4-nitroquinoline-N-oxide; ɣH2AX—H2A histone family member X; apoM—apolipoprotein M; Bcl-2—B-cell lymphoma 2; C2C12—mouse myoblast cell line; CAT—catalase; Cdk—cyclin-dependent kinase; CREB—cAMP-response-element-binding protein; DAPK1—death-associated protein kinase 1; ERK1/2—extracellular signaling-regulated kinase; FOXO3—forkhead box O3; G6PD—glucose-6-phosphate dehydrogenase; GCLC—glutamate-cysteine ligase catalytic subunit; GSR—glutathione reductase; HepG2—human liver cancer cell line; hMSCs—human mesenchymal stem cell line; HT-29—colon carcinoma cell line; KEAP1—Kelch-like ECH-associated protein 1; LNCaP—androgen-sensitive human prostate adenocarcinoma cell line; LVDP—left ventricular developed pressure; NHDF—neonatal normal human dermal fibroblasts; NHEK—neonatal normal human epidermal keratinocytes; NK—human natural killer cells; NPCs—neural progenitor cells; NQO1—NAD(P)H dehydrogenase [quinone] 1; Nrf2—nuclear factor erythroid 2-related factor 2; ODC—ornithine decarboxylase; p21^Cip1^ cyclin-dependent kinase inhibitor 1; p27^Kip1^—cyclin-dependent kinase inhibitor 1B; PSA-NCAM—polysialylated neuronal cell adhesion molecule; p38—mitogen-activated protein kinase; PKC—protein kinase C; SAC—spindle assembly checkpoint; SIRT—sirtuin; SOX2—transcription factor (sex-determining region Y-box 2), TrkA—tropomyosin receptor kinase A. Resveratrol used in cited experiments was of commercial origin.

**Table 3:** Phytochemicals exhibiting biphasic concentration/dose-responses.

Compound *	Model	Concentration	Effect	Mechanism	Refs
Arctigenin	K-562	~27, 54 μM	↑Mcl-1mRNA		[85]
		~107 μM	↓Mcl-1mRNA		
Berberine	B16-F10,	1.25–5.00 μM	↑proliferation	↑MAPK/ERK1/2 ↑PI3K/AKT	[70]
	MDA-MB-231,	10–80 μM	↓proliferation		
	MDA-MB-468,				
	MCF-7, LS-174				
	PC-12	0.1–1.0 μM	↑viability	↑PI3K/AKT/Bcl-2	[71]
		2–64 μM	↓viability		
Caffeic acid	male F344 rats	0.14%	↓proliferation	↓epithelial cells, S-phase cells	[90]
		0.40, 1.64%	↑proliferation	↑epithelial cells, ↓S-phase cells in forestomach	
(+) Catechin, rutin	*Salmonella typhimurium*/2-AAF	0.01–0.60 mM	↓mutagenicity		[35]
		1.2, 0.8 mM	↑mutagenicity		
Cynarin	FSF-1,	1–50 µM	↑viability	↑HO-1 activity	[89]
		75–500 µM	↓viability		
	hTERT-MSC	1–00 µM	↑viability	↑HO-1 activity	[89]
		75–500 µM	↓viability		
EGCG	*Caenorhabditis elegans*	50–300 µM	↑lifespan	↑ROS; ↑AMPK/SIRT1/FOXO	[87]
		800–1000µM	↓lifespan		
Falcarinol, Falcarindiol Isolated from carrot roots	primary myotube culture/H_2_O_2_	1.6–25.0 μM	↑ROS production	↑*GPx, *↓*Hsp70, HO-1*	[72]
		50, 100 μM	↓ROS production	↓*GPx, *↑*Hsp70, HO-1*	
Falcarindiol isolated from carrot roots	primary	0.61–9.80 nM	↑viability		[72]
	myotube culture	2.5–5.0 μM	↓viability		
	pBMEC	~0.04–0.20 μM	↑proliferation		[73]
		~4–41 μM	↓proliferation		
	CaCo-2	1–10 μM	↑proliferation	↓caspase-3, DNA breakage	[74]
			↓apoptosis		
		>20 μM	↓proliferation	↑caspase-3, DNA breakage	
			↑apoptosis		
Glyceollin I isolated from soybean	*Saccharomyces* *cerevisiae*	10–100 nM	↑CLS		[82]
		>1 μM	↓CLS		
Luteolin	MCF-7	1–10 μM	↑viability		[77]
		30–1000 μM	↓viability		
	HepG2	<35 μM	↑LC3-II		[78]
		~105 μM	↓LC3-II		
	*Salmonella typhimurium*/2-AAF	0.006 mM	↑mutagenicity		[35]
		1.2 mM	↓mutagenicity		
Nanteine isolated from* Ocotea macrophilla*	synaptosomal membranes	50 μM, 0.3 mM	↑K+ -p-NPPase activity		[84]
		>0.75 mM	↓K+ -p-NPPase activity		
Naringenin	*Drosophila melanogaster*	200, 400 μM	↑lifespan	↑pupae formation	[75]
		600, 800 μM	↓lifespan	↓pupae formation	
Naphazarin	*Caenorhabditis* *elegans*	50–500 μM	↑lifespan	↑skn-1	[86]
		1000 µM	↓lifespan		
Panaxatriol saponins isolated from* Panax notoginseng*	PC-12	0.03–1.00 mg/ml	↑proliferation		[88]
		4 mg/ml	↓proliferation		
	PC-12 /6-OHDA	0.03–2.00 mg/ml	↑viability	↑PI3K/AKT/mTOR ↑AMPK/SIRT1/FOXO3	
		4 mg/ml	↓viability		
Plumbagin	*Caenorhabditis elegans*	1–45 μM	↑lifespan	↑skn-1	[86]
		100 μM	↓lifespan		
Rosmarinic acid	*Caenorhabditis* *elegans*	100–300 µM	↑lifespan	↑*hsp*	[34]
		600 µM	↓lifespan		
Rutin	*Drosophila melanogaster*	200, 400 μM	↑lifespan	↑longevity associated genes	[76]
		600, 800 μM	↓lifespan		
Salvianolic acid B	BMSCs	~4–111 μM	↑metabolic activity, ALP activity		[81]
		~223 μM	↓metabolic activity, ALP activity		
Sulforaphane	T24, HepG2, Caco-2	1–5 μM	↑proliferation	↑RAS, RAF, MEK, ERK, PI3K, AKT and Nf-kB, FOXO Nrf2 pathways	[63]
		10–40 μM	↓proliferation		
	T24	2.50, 3.75 μM	↑migration		
		5–40 μM	↓migration		
	HUVEC, PVC	2.5–5.0 μM	↑angiogenesis	↑tube formation	
		10, 20 μM	↓angiogenesis	↓tube formation	
Isolated from* Brassica oleracea*	MSCs	0.25, 1.00 μM	↑proliferation		[66]
		20 μM	↓proliferation		
		<5 μM	↓apoptotic cells		
		20 μM	↑apoptotic cells		
		0.25, 1.00 μM	↓senescence cells		
		5, 20 μM	↑senescence cells		
		0.25 μM	↓ROS production		
		20 μM	↑ROS production		
Commercial source	MCF-7, HHL-5, HepG2, lymphoblastoid cells	<5 μM	↑proliferation		[67,68,69]
		>5 μM	↓proliferation		
	lymphoblastoid cells	0.5–5.0 μM	↑GSH		[69]
		10 μM	↓GSH		
Umbelliprenin isolated from* Ferula szowitsiana*	Jurkat T-CLL	10, 25 μM	↑apoptosis		[83]
		50, 100 μM	↓apoptosis		
Z-ligustilide isolated from* Ligusticum chuanxiong*	PC-12/ OGD	1–25 μM	↑viability, ↓apoptosis	↑HO-1 and Nrf2 translocation	[79]
		50 μM	↓viability, ↑apoptosis		
	*Spodoptera litura* larvae	0.1–0.5 mg/g diet	↑GST, AChE, CYP, CES activities	↑*GSTS1, CYP4S9, CYP4M14*	[80]
		1, 5 mg/g diet	↓GST, AChE, CYP activity	↓*GSTS1, CYP4S9, CYP4M14*	

* If the source of the compound was not specified it was obtained commercially; ↑ = increase, ↓ = decrease; 2-AAF—2-Acetylaminofluorene; 6-OHDA—6-hydroxydopamine; AChE—acetylcholinesterase; AKT—protein kinase B; ALP—alkaline phosphatase; B16-F10—murine melanoma cell line; BMSCs—bone marrow-derived mesenchymal stem cells; CaCo-2—human colon cancer cell line; CES—carboxylesterase; CLS—chronologic life span; CYP—cytochrome P450; CYP4M14 (4S9)—cytochrome P450 4M14 (4S9); EGCG—epigallocatechin-3-gallate; FSF-1—human skin fibroblasts; GPx—glutathione peroxidase; GST—glutathione S-transferase; GSTS1—glutathione S transferase S1; HHL-5—human normal liver cell line; HO-1—heme oxygense 1; Hsp70—heat shock protein; HepG2—human liver cancer cell line; HUVEC—human umbilical vein endothelial cells; Jurkat T-CLL—Jurkat T-cell lymphocyte leukemia cells; K562—immortalized cell line derived from human leukemia; K+ -p-NPPase activity—K+ -p- nitrophenylphosphatase; LC3— microtubule-associated protein 1A/1B-light chain 3; LS-174—human colon cancer cell line; MDA-MB-231, MDA-MB-468, MCF-7MCF-7—human breast carcinoma cell lines; MSCs—mesenchymal stem cell line; OGD—oxygen-glucose deprivation; pBMEC—primary bovine mammary epithelial cells; PC-3—human prostatic carcinoma cell line; PC-12—phaeochromocytoma cell line; PI3K—phosphatidylinositol 3-kinase; PVC—pericytes; skn-1—cap’n’collar transcription factor; T24—bladder cancer cell line; hTERT-MSC—human normal telomerase-immortalized mesenchymal stem cells.

## Abbreviations

2-AAF	2-Acetylaminofluorene
4NQO	4-nitroquinoline-N-oxide
6-OHDA	6-Hydroxydopamine
AChE	Acetylcholinesterase
AFB1	Aflatoxin B1
AKT	Serine/threonine protein kinase
ALP	Alkaline phosphatase
apoM	Apolipoprotein M
b.w.	Bodyweight
B16-F10	Murine melanoma cell line
Bcl-2	B-cell lymphoma 2
BER	Berberine
BMSCs	Bone marrow-derived mesenchymal stem cells
C2C12	Mouse myoblast cell line
CA	Caffeic acid
CaCo-2	Human colon cancer cell line
CD203c	Basophil-specific ectoenzyme E-NPP3
CD63	Tetraspan transmembrane protein family
CES	Carboxylesterase
CIN	Chromosomal instability
CLS	Chronologic life span
CYN	Cynarin
CYP	Cytochrome P450
CYP1A2	Cytochrome P450 1A2
DAI	Daidzein
ER	Estrogen receptor
ERK	Extracellular signal-regulated kinase protein-serine/threonine kinase
fMLP	Bacterial formyl peptide N-formylmethionine-leucine-phenylalanine
FSF-1	Human skin fibroblasts
GEN	Genistein
GIR	Genotoxic inhibition rate
GPx	Glutathione peroxidase
GSH	Reduced glutathione
GSSG	Oxidized glutathione
GST	Glutathione S-transferase
HCT-116	Colon carcinoma cell lines
HepG2	Human liver cancer cell line
HHL-5	Human normal liver cell line
HO-1	Heme oxygenase-1
Hsp70	70 kDa heat shock protein
HT-29	Colon carcinoma cell lines
hTERT-MSC	Human normal telomerase-immortalized mesenchymal stem cells
HUVEC	Human umbilical vein endothelial cell line
ISL	Isoliquiritigenin
Jurkat T-CLL	Jurkat T-cell lymphocyte leukemia cells
K+ -p-NPPase activity	K+ -p- nitrophenylphosphatase
K562	Immortalized cell line derived from human leukemia
KS483	Murine osteoprogenitor cell line
LC3-II	Microtubule-associated protein 2 light chain 3
LNCaP	Androgen-sensitive human prostate adenocarcinoma cell line
LoVo	Human colon adenocarcinoma cell line
LS-174	Human colon cancer cell line
LVDP	Left ventricular developed pressure
MAPK	Mitogen-activated protein kinase
MCF-7	Human breast adenocarcinoma cell line
MDA-MB-231, MDA-MB-468, MCF-7MCF-7	Human breast carcinoma cell lines
MeIQ	2-amino-3,4-dimethylimidazo [4,5-f]quinoline
MG-63	Human osteoblast-like cells
MMPs	matrix metalloproteinases
MN	Markers micronuclei
MSCs	Mesenchymal stem cell line
NHDF	Neonatal normal human dermal fibroblasts
NHEK	Neonatal normal human epidermal keratinocytes
NK	Human natural killer cells
NPB	Nucleoplasmic bridge
NPCs	Neural progenitor cells; ODC - ornithine decarboxylase
OGD	Oxygen-glucose deprivation
pBMEC	Primary bovine mammary epithelial cells
PC-12	Phaeochromocytoma cell line
PC-3	Human prostatic carcinoma cell line
PCNA	Proliferating cell nuclear antigen
PGE2	Prostaglandin E2
PI3K	Phosphoinositide 3-kinase
PKC	Protein kinase C
PMA	Phorbol myristate acetate
PPARγ	Peroxisome proliferator-activated receptor γ
PTS	Panaxatriol saponins
PVC	Pericytes
QER	Quercetin
RA	Rosmarinic acid
RAW 264.7	Murine macrophage cell line
RES	Resveratrol
ROS	Reactive oxygen species
RWPE-1	Nontumorigenic human prostate epithelial cells
SCC-25	Oral squamous carcinoma cell line
SFN	Sulforaphane
SKN-1	Transcription factor skinhead-1
SOX2	Transcription factor (sex determining region Y-box 2
T24	Bladder cancer cell line
T-47D, T	Human breast cancer cell lines
TIMP-2	Tissue inhibitor of metalloproteinase-2
TRAMP-FVB	Transgenic adenocarcinoma of mouse prostate model
UtLM	Human uterine leiomyoma
UtSMCs	Uterine smooth muscle cells
